# Choline alphoscerate: insights between acquired certainties and future perspectives

**DOI:** 10.3389/fnagi.2025.1613566

**Published:** 2025-08-06

**Authors:** Giovanni Biggio, Claudio Mencacci

**Affiliations:** 1 Department of Life and Environmental Sciences, University of Cagliari, Cittadella Universitaria di Monserrato, Cagliari, Italy; 2 Institute of Neuroscience, CNR, Cittadella Universitaria di Monserrato, Cagliari, Italy; 3 Department of Neuroscience and Mental Health, ASST Fatebenefratelli Sacco, Milan, Italy

**Keywords:** aging, choline alphoscerate, cognitive dysfunction, mild cognitive impairment, sleep disorders

## Abstract

While mild cognitive impairment (MCI) is a risk factor for dementia, it is currently impossible to predict which patients will go on to develop dementia or Alzheimer’s disease. Given the projected global increase in dementia due to an increasingly aging population, there is an urgent need to develop pharmacological therapies to reduce symptoms of MCI, and to help delay its possible progression to dementia. Choline alphoscerate is a cholinergic precursor naturally found in the brain that has been identified as an essential nutrient and is available as a prescription drug. While the efficacy of choline alphoscerate on cognitive function is well established in patients with MCI, Alzheimer’s disease, and cognitive impairment of vascular origin, emerging evidence suggests that it has neuroprotective effects against *β*-amyloid injury and may be useful as a preventive therapy against development of Alzheimer’s disease in patients with MCI. Recent data also show that choline alphoscerate may be effective against non-cognitive symptoms of MCI (e.g., depression, anxiety, irritability, aggression, and apathy). Here we review pharmacological and clinical evidence regarding choline alphoscerate in order to highlight its usefulness in patients with MCI. The potential role of choline alphoscerate in promoting healthy sleep architecture is also explored.

## Graphical abstract

**Figure:**
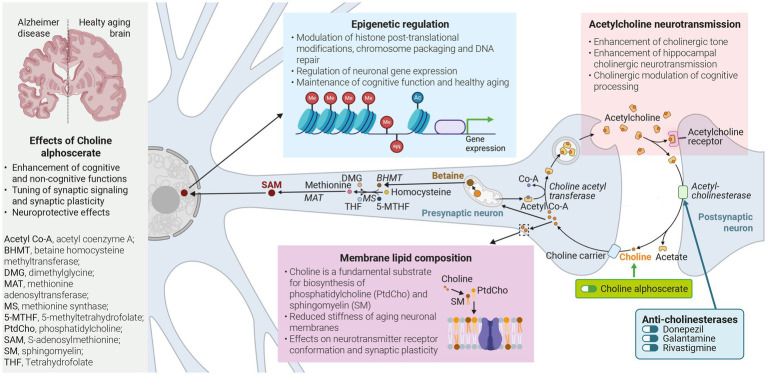

## 1 Introduction

Affecting up to 27% of people aged 65 years and older (Scafato et al., 2010; Anderson, 2019; Jia et al., 2020; Bai et al., 2022), mild cognitive impairment (MCI) is considered a transitional stage between healthy aging and dementia (Jia et al., 2020; Bai et al., 2022; Han and Chul Youn, 2022; Morozova et al., 2022). Some individuals may notice a decline in cognitive function before being diagnosed with MCI, but show no objective impairment by neuropsychological tests and are generally considered clinically healthy, with normal daily functioning and independence (Jessen et al., 2020). This preclinical condition is known as subjective cognitive decline (SCD), which has been linked to an increased risk of future objective cognitive decline (Jessen et al., 2020). In addition to cognitive symptoms, non-cognitive symptoms in MCI include depression, anxiety, irritability, aggression, and apathy (Mougias et al., 2023). Several forms of MCI have been proposed, with varying clinical outcomes: degenerative (onset low and gradual), vascular (in patients with higher vascular risk), and anxiety and depression (in patients with a history of psychiatric syndromes) (Petersen, 2016). Also, new-onset cognitive impairment, associated with abnormal brain metabolism, has often been reported after coronavirus disease 2019 (COVID-19) (Beretta et al., 2023; Ferrucci et al., 2023).

Dementia is commonly classified as either dementia of primary origin (i.e., dementia of degenerative origin such as that associated with Alzheimer’s disease or Parkinson’s disease), dementia of secondary origin (i.e., dementia that is a consequence of conditions that cause cognitive impairment as a secondary effect such as vascular dementia), or mixed dementia (e.g., Alzheimer’s disease with simultaneous vascular dementia; Kabasakalian and Finney, 2009; Bello and Schultz, 2011).

While MCI is a risk factor for dementia (Knopman et al., 2021), not all patients with MCI go on to develop dementia (Mitchell and Shiri-Feshki, 2009; Bai et al., 2022; Han and Chul Youn, 2022) or Alzheimer’s disease (Mitchell and Shiri-Feshki, 2009); however, it is currently impossible to predict which patients with MCI will advance to Alzheimer’s disease (Bateman et al., 2012; Morozova et al., 2022). Other known risk factors for the development of Alzheimer’s disease include age >65 years, presence of the epsilon 4 allele of the apolipoprotein E (apoE) gene, female sex, diabetes mellitus, arterial hypertension, smoking, obesity, low levels of high-density lipoprotein cholesterol, hearing loss, traumatic brain damage, depression and social isolation, low physical activity, alcohol abuse, and air pollution (Knopman et al., 2021).

Chronic stress often manifests as depression/apathy and insomnia, and has been associated with cognitive impairment and Alzheimer’s disease, among other disorders (McEwen, 2006; Groeneweg-Koolhoven et al., 2017; Hamdy et al., 2018; Biella et al., 2019; Baek et al., 2020). The hippocampus, an area in the brain that is responsible for cognitive function, is known to adapt in response to stress (McEwen, 2006). Chronic stress and alterations in sleep patterns often result in reduction of neuronal trophism in the medial prefrontal cortex, and is linked with cognitive impairment and depression (McEwen et al., 2016). Furthermore, the presence of apathy and other neuropsychiatric disorders in patients with MCI may be a risk factor for the development of dementia (Ellwardt et al., 2015; Tomioka et al., 2015;van Dalen et al., 2018a; van Dalen et al., 2018b; Roberto et al., 2021).

Given an increasingly aging population globally and projected increase in associated dementia (Livingston et al., 2017; GBD 2019 Dementia Forecasting Collaborators, 2022), there is an urgent need to develop pharmacological therapies to reduce the symptoms of MCI and to delay the possible progression to dementia (Sagaro et al., 2023).

Choline alphoscerate is a choline-containing phospholipid naturally found in the brain, that has been identified as an essential nutrient (Kansakar et al., 2023; Sagaro et al., 2023). Due to its cognition-enhancing capabilities by counteracting reduced cholinergic tone, which is the basis of cognitive dysfunction, choline alphoscerate (Delecit®) is a prescription drug that is a useful treatment for cognitive impairment in Alzheimer’s disease, and other types of MCI and adult-onset dementias (Sagaro et al., 2023). Results of systematic reviews and meta-analyses suggest that choline alphoscerate not only improves cognitive performance but may also reduce cognitive decline (Parnetti et al., 2001; Sagaro et al., 2023). Indeed, *in vitro* data suggest that choline alphoscerate has neuroprotective effects against *β*-amyloid injury (Catanesi et al., 2020).

The aim of this narrative review is to discuss the pharmacological and clinical evidence regarding choline alphoscerate in order to highlight its usefulness in patients with MCI, including a potential protective role in β-amyloid (Aβ)1–42-induced microglia activation. This review will also evaluate the potential role of choline alphoscerate in promoting healthy sleep architecture.

## 2 Methods

Identification of supporting evidence for this narrative review, using structured literature searching of the PubMed database and *ad hoc* online searches, was conducted on 18 July 2024. The PubMed search terms included “choline alphoscerate” or “choline alphoscerate,” in combination with “pharmacology” or disease-related terms such as “mild cognitive impairment,” “sleep,” “apathy.” No other limits (e.g., time period, language, reviews) were applied to the searches. Search results were filtered for relevant preclinical and clinical studies. In addition, content for the article was identified based on the authors’ knowledge of the therapeutic area.

## 3 Choline alphoscerate as a source of choline for the organism

**Figure 1:**
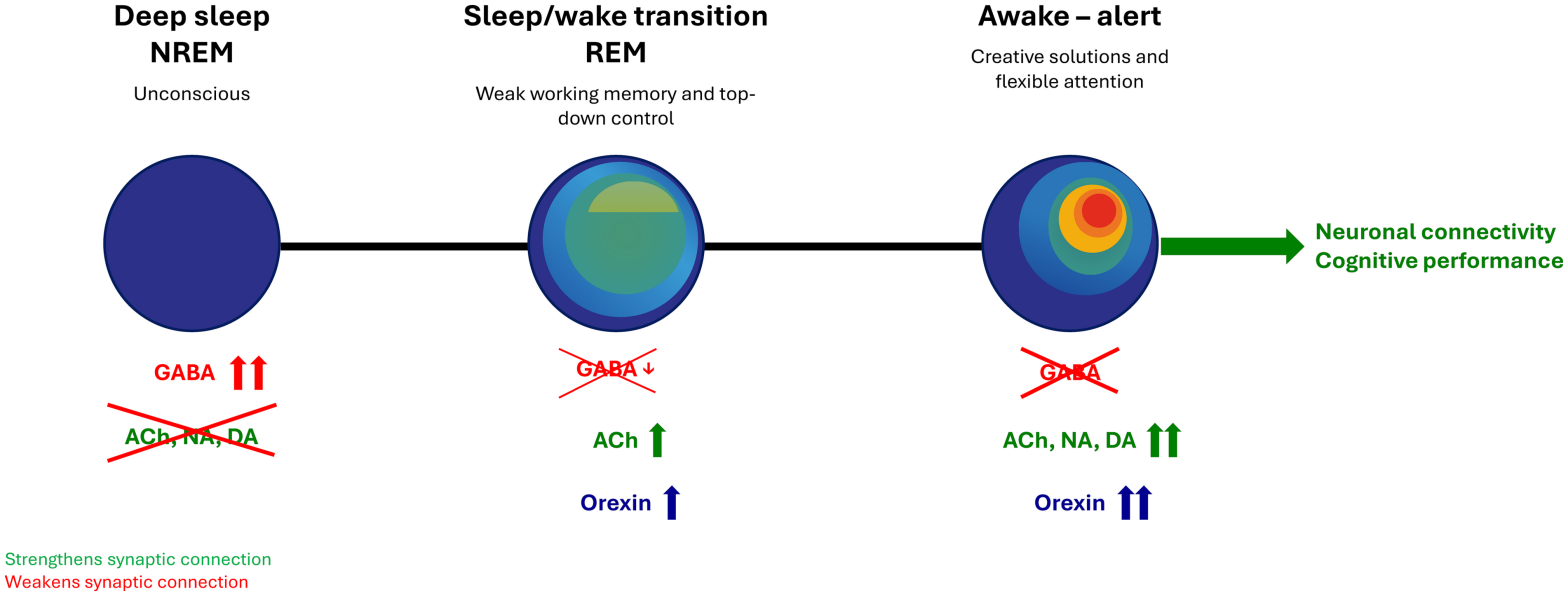
Metabolism and physiology of choline (Goh et al., 2021). Permission to reproduce this figure (Goh et al., 2021) obtained from the license holder, The American Chemical Society.

Choline is an essential nutrient for the body, required especially for the functioning of the brain and nervous system (National Institutes of Health, 2022; Gallo and Gámiz, 2023). It is implicated in neurotransmission, cell-membrane signaling, lipid transport, and methyl-group metabolism (Goh et al., 2021; National Institutes of Health, 2022; Gallo and Gámiz, 2023). In the brain, choline is a precursor of various metabolites, including the neurotransmitter acetylcholine, membrane phospholipids (i.e., phosphatidylcholine and sphingomyelin) and the methyl donor betaine (Figure 1; Goh et al., 2021; National Institutes of Health, 2022). Among these, acetylcholine is a crucial neurotransmitter involved in cognitive function, with acetylcholine deficiency implicated in the cognitive dysfunction that characterizes patients with dementia (Hampel et al., 2018).

Choline can be naturally synthesized in the body, mostly as phosphatidylcholine (National Institutes of Health, 2022; Gallo and Gámiz, 2023). Estrogen activates the gene that catalyzes choline biosynthesis (Goh et al., 2021; National Institutes of Health, 2022), which may account for between-sex differences observed in the risk of development of cognitive impairment and Alzheimer’s disease (Li and Singh, 2014; Rettberg et al., 2014). However, the amount of choline produced by the body is generally insufficient to meet human needs; thus, the diet is an important alternate source of choline (Hollenbeck, 2012; National Institutes of Health, 2022). The United States National Academy of Medicine (NAM) and the European Food Safety and Authority (EFSA) have specified adequate intake values for choline (Goh et al., 2021). However, it is also important to understand individual differences in choline bioavailability and utilization caused by genetic, age, sex, and ethnic differences, as well as the effects of dietary preferences, gut enterotype, intestinal absorption, and lifestyle.

Choline alphoscerate (C8H20NO6P) is a cholinergic drug that is widely used for enhancement of cholinergic transmission (Kansakar et al., 2023). Although also used as a food supplement, choline alphoscerate is available as a prescription drug, and therefore subject to tight regulation and rigorous testing to provide evidence of effectiveness and safety (Hathcock, 2001; Dwyer et al., 2018). In contrast, food supplements are self-regulated by the manufacturer, and proof of effectiveness and safety are not required except where health benefits are being claimed.

**Figure 2:**
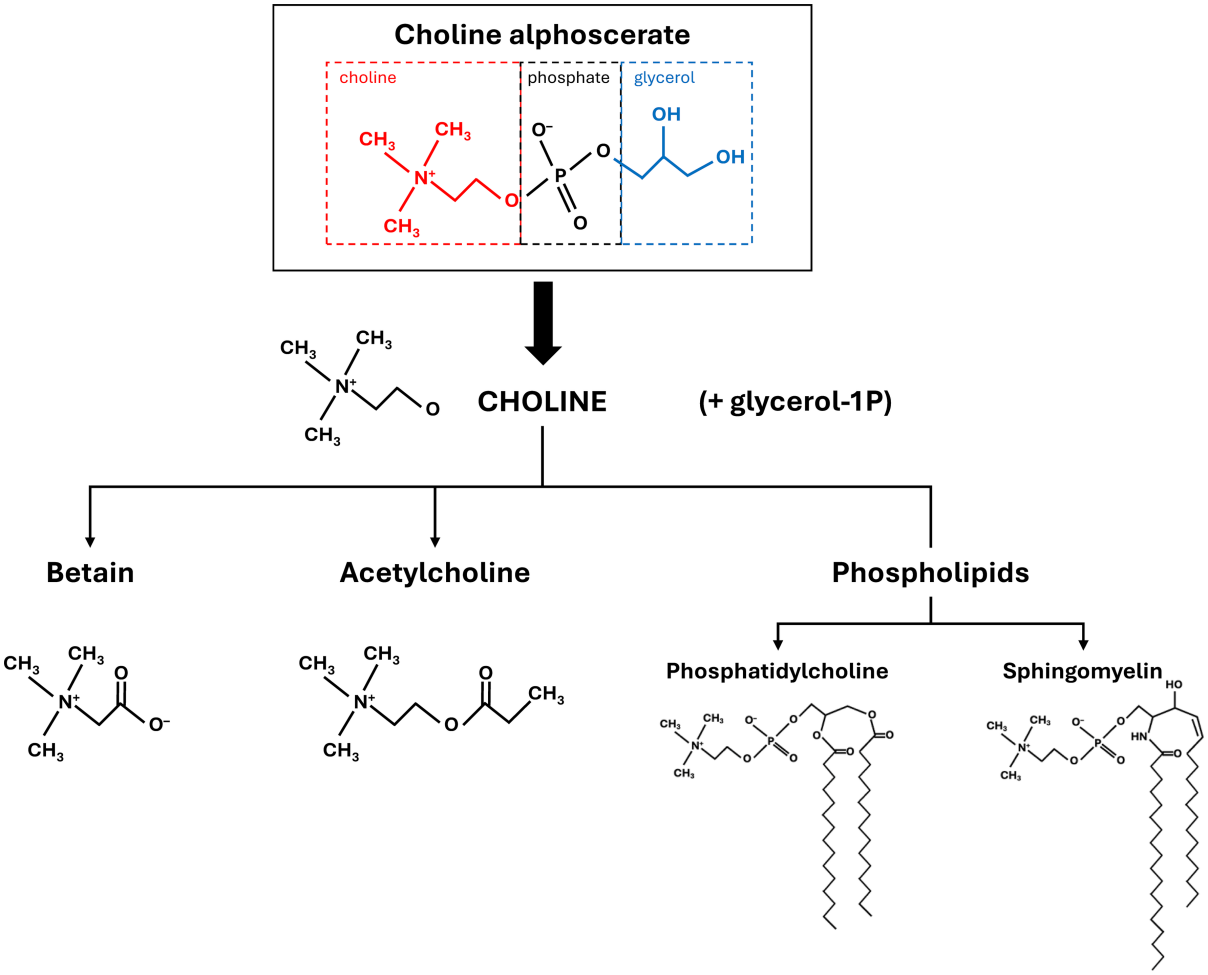
The role of choline-containing compounds [i.e., choline alphoscerate, citicoline (CDP-choline)] in acetylcholine synthetic pathways (Amenta et al., 2001; Traini et al., 2013). Permission to reproduce this figure obtained from license holder, Elsevier Science Ac-CoA, acetyl coenzyme A; ACh, acetylcholine; ADP, adenosine diphosphate; ATP, adenosine triphosphate; CDP, cythidin diphosphate; ChAT, choline acetyltransferase; ChK, choline kinase; CoA, coenzyme A; CTP, cythidin triphosphate; GDP, glyceryl-phosphorylcholine diesterase; P, phosphate; PAT, phosphocholine acydil transferase.

Due to its high choline content (41% by weight) and its ability to cross the blood–brain barrier, choline alphoscerate is a useful source of choline (Kansakar et al., 2023). Compared with citicoline (CDP-choline), an alternative source of choline, choline alphoscerate is rapidly and directly metabolized into the active form of choline that is able to enhance release of the neurotransmitter acetylcholine and brain-derived neurotropic factor after administration; in contrast, citicoline is an indirect substrate because it requires additional metabolic steps to produce choline and, therefore, acetylcholine (Figure 2; Traini et al., 2013; Kansakar et al., 2023). Mean increases in free plasma choline levels are greater after administration of choline alphoscerate than after citicoline (25.8 versus 13.1 μmol/L) (Gatti et al., 1992). The above mentioned cholinergic precursors (choline alphoscerate and citicoline) represent one the first approaches attempting to relief cognitive impairment and they are still used today due to their demonstrated efficacy. However, is important to consider that other form of choline-containing phospholipids (alone or in combination with colinesterase inhibitors) failed to show significant efficacy in terms of cognitive improvement in controlled clinical trials (Kansakar et al., 2023).

**Figure 3:**
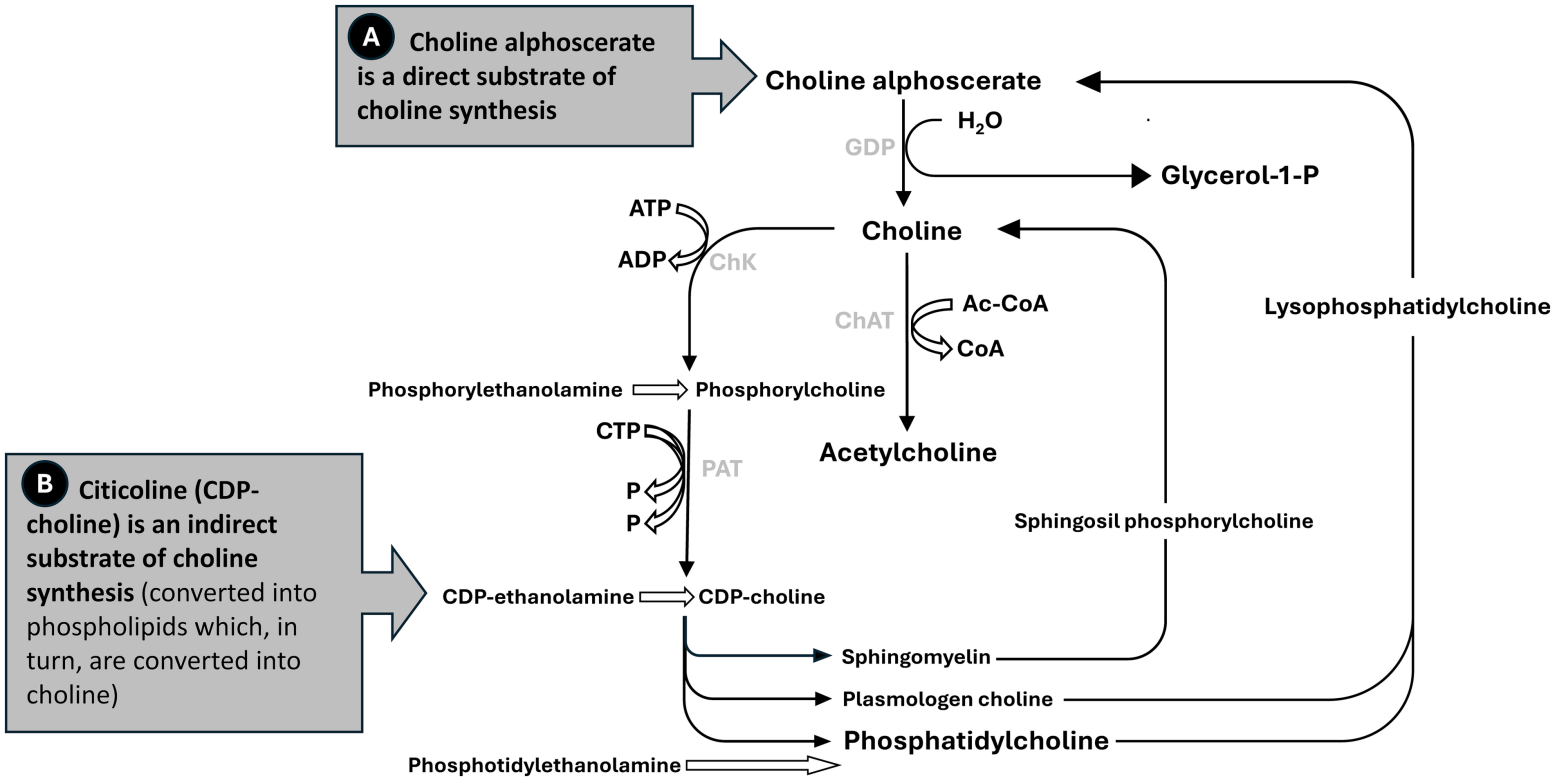
Metabolism of choline alphoscerate.

In this context, choline alphoscerate, as well as being a valuable source of choline for acetylcholine synthesis, also provides choline for phospholipid biosynthesis and betaine formation (Figure 3; Kansakar et al., 2023). Choline alphoscerate is also a direct substrate for choline synthesis, with metabolism of choline alphoscerate providing both free choline for acetylcholine synthesis and phospholipids as components of nerve cells (Traini et al., 2013; Roy et al., 2022). The roles of each of these substances in the brain are briefly described below.

### 3.1 Acetylcholine

Acetylcholine is one of the most important neurotransmitters in the brain (Hampel et al., 2018). Neurons that synthesize acetylcholine are located within the basal forebrain, with axonal projections throughout the cholinergic system (Perry et al., 1999; Cools and Arnsten, 2022). The cholinergic system plays a crucial role in neuroimmune communication, as it contains the nucleus basalis of Meynert (nbM) and the medial septum that provide primary cholinergic innervations to the cerebral cortex and hippocampus in support of memory, attention, executive functions, and aversive learning (Chaudhary et al., 2022). Pathological changes of the nbM disrupt limbic acetylcholine and induce acetylcholine deficiency, which is thought to play a prominent role in cognitive deficits of various dementia syndromes (i.e., the cholinergic hypothesis), including Alzheimer’s disease (Hampel et al., 2018; Chaudhary et al., 2022; Lee and Hung, 2022). Thus, treatments that improve cholinergic function are crucial for the management of symptoms in patients with Alzheimer’s disease (Hampel et al., 2018; Chaudhary et al., 2022; Lee and Hung, 2022).

Furthermore, during the waking state, acetylcholine helps coordinate and fine-tune brain activity in response to external and internal events (Cools and Arnsten, 2022). Cholinergic nuclei are also involved in controlling sleep versus waking states (Cools and Arnsten, 2022).

### 3.2 Phospholipids

Phospholipids are major constituents of neuronal membranes (Binotti et al., 2021). Phosphatidylcholine (32.8%), phosphatidylethanolamine (35.6%), phosphatidylinositol (2.6%), and sphingomyelin are the main phospholipids present in human membranes (Binotti et al., 2021). Of these, choline metabolism is involved in the creation of phosphatidylcholine and sphingomyelin (Goh et al., 2021; National Institutes of Health, 2022). Sphingolipids regulate neurotransmitter receptor conformation (within membranes directly), function, and trafficking (Egawa et al., 2016). Phospholipids are also involved in synaptic plasticity, essential for information processing by the brain and adaptation to changing external and internal stimuli (García-Morales et al., 2015). In the aging brain, changes in synaptic membrane lipids are associated with decreased neuroplasticity and loss of neuronal function (Egawa et al., 2016; Skowronska-Krawczyk and Budin, 2020).

### 3.3 Betaine

Also known as trimethyglycine, betaine is a naturally occurring short-chain amino acid derivative that can be found in some foods, which can also be synthesized in the body via choline metabolism (Arumugam et al., 2021). Betaine has many functions, including inhibition of nuclear factor kappa B (NF-κB) activity, reduction in inflammatory activation, endoplasmic reticulum stress, and apoptosis, regulation of energy metabolism, and anti-cancer effects (Zhang and Tang, 2023). Importantly, betaine is also known to provide neuroprotective effects through increasing silent information regulator 1 (SIRT1) activity (Zhang and Tang, 2023). SIRT1 is a group III histone deacetylase involved in many functions, including gene transcription, inflammatory and autoimmune responses, energy metabolism, cell aging, regulation of metabolic homeostasis, and tumorigenesis (Zhang and Tang, 2023). SIRT1 is widely expressed in the brain, mostly in the nucleus of neurons (Zhang and Tang, 2023).

With respect to Alzheimer’s disease, betaine is an important methyl donor in the methionine cycle, critical in epigenetic mechanisms (Hollenbeck, 2012; Arumugam et al., 2021; Kansakar et al., 2023; Zhang and Tang, 2023). Histone post-translational modifications, involved in regulation of transcription activation or inactivation, chromosome packaging and DNA repair, play a role in controlling the lifespan (Zhang and Tang, 2023). However, while SIRT1-associated maintenance of epigenomic integrity and appropriate DNA methylation patterns can extend the lifespan, SIRT1 expression decreases with age (Zhang and Tang, 2023). Betaine intake has been shown to prevent the development of cognitive impairment in a mouse model of Alzheimer’s disease by preventing decreased hippocampal expression of SIRT1 (Ibi et al., 2022).

## 4 The role of choline alphoscerate in sleep

**Figure 4:**
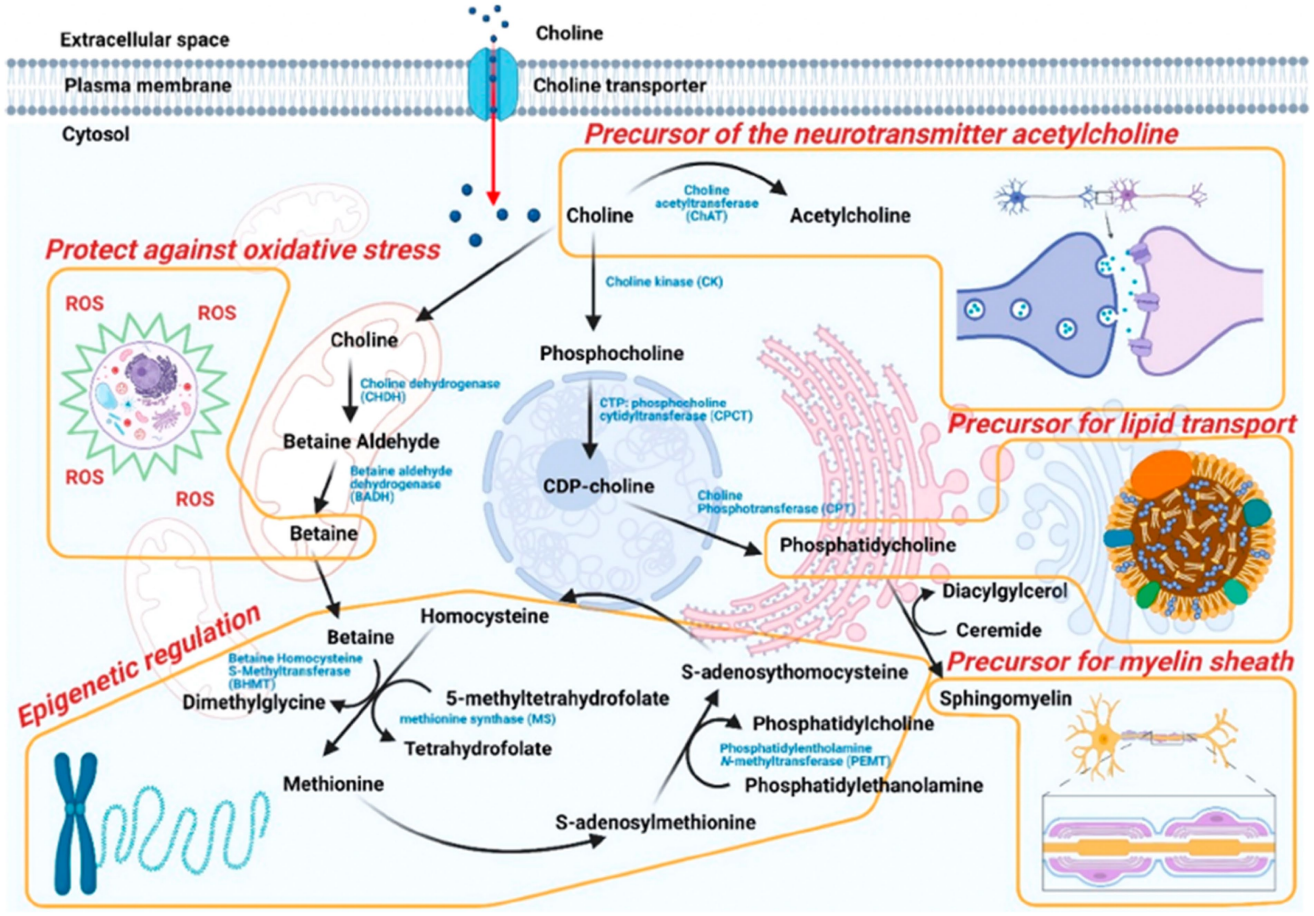
With increasing neuronal arousal, cognitive performance also increases due to activation of orexin and excitatory neurotransmitters. Green indicates strengthening of synaptic connections, whereas red denotes weakening of synaptic connections. Ach, acetylcholine; DA, dopamine; GABA, gamma-aminobutyric acid; NA, noradrenalin; NREM, non-rapid eye movement; REM, rapid eye movement.

Choline also plays a role in sleep, which may impact memory and cognitive function. Cholinergic neurons are activated during rapid eye movement (REM) sleep, or dreaming sleep; REM is triggered by the firing and release of acetylcholine from pedunculopontine cholinergic neurons (Perry et al., 1999; Cools and Arnsten, 2022). With increasing neuronal arousal, cognitive performance increases due to activation of orexin and excitatory neurotransmitters (e.g., acetylcholine, noradrenalin, dopamine; Figure 4). It is also known that age-related decrease in memory retention is associated with impaired mechanisms of sleep-dependent memory consolidation (Mander et al., 2013).

Thus, ensuring adequate levels of acetylcholine in the brain may be useful in restoring sleep patterns in the aging brain, as well as possible prevention of sleep disorders, depression, and/or stress. In this regard, and as mentioned previously, choline alphoscerate is an important cholinergic precursor useful for improving reduced cholinergic tone in patients with dementia (Carotenuto et al., 2022), and is a precursor of phospholipids (Traini et al., 2013), which help to maintain the plasticity of neuronal membranes (García-Morales et al., 2015).

## 5 Clinical studies of choline alphoscerate

### 5.1 Efficacy of choline alphoscerate on cognitive symptoms

The effects of choline alphoscerate on cognitive impairment are well characterized. Table 1 outlines clinical studies of evaluating the cognitive efficacy of choline alphoscerate in various types of dementia. In short, choline alphoscerate has demonstrated improved cognitive function in patients with MCI or dementia and reduced progression of cognitive deterioration in patients with Alzheimer’s disease, when administered as monotherapy or in combination with donepezil (Parnetti et al., 2001; Parnetti et al., 2007; Traini et al., 2013). The reproducibility of the findings of these trials was confirmed in a recent controlled study that showed that the combination of donepezil with choline alphoscerate enhanced cognitive function more effectively than donepezil alone or donepezil in combination with other nootropic agents (Lee and Kim, 2024).

**Table 1:** Summary of clinical studies investigating the efficacy of choline alphoscerate on cognitive symptoms in neurodegenerative, vascular, and mixed types of dementia.

Study design	Origin of cognitive impairment^a^		
	Neuro degenerative	Vascular	Mixed^b^
Controlled studies	RCT, IM choline alphoscerate 1,000 mg/day vs. oxiracetam for 3 months (Abbati et al., 1991)	RCT, OL, IM choline alphoscerate 1,000 mg/day vs. citicoline 1,000 mg/day for 90 days (Di Perri et al., 1991)	SB, oral choline alphoscerate 1,200 mg/day vs. placebo for 3 months (Vezzetti and Bettini, 1992)
	RCT, DB, oral choline alphoscerate plus donepezil vs. donepezil plus placebo for 3 years (Amenta et al., 2012; Amenta et al., 2014; Amenta et al., 2016; Traini et al., 2020)	RCT, OL, IM choline alphoscerate 1,000 mg/day vs. citicoline 1,000 mg/day for 90 days (Frattola et al., 1991)	
	RCT, oral choline alphoscerate 1,200 mg/day plus donepezil 10 mg/day vs. donepezil 10 mg/day plus placebo for 24 months (Carotenuto et al., 2022)	RCT, OL, IM choline alphoscerate 1,000 mg/day vs. citicoline 1,000 mg/day for 90 days (Muratorio et al., 1992)	
	RCT, DB, oral choline alphoscerate 1,200 mg/day vs. placebo for 6 months (De Jesus Moreno Moreno, 2003)	RCT, OL, oral choline alphoscerate 1,200 mg/day vs. oxiracetam 1,600 mg/day for 6 months (Paciaroni and Tomassini, 1993)	
	OL, IV choline alphoscerate 1,000 mg/day vs. IV piracetam 2000 mg/day for 10 days (Levin et al., 2011)		
	RCT, oral choline alphoscerate 1,200 mg/day vs. acetyl-L-carnitine 1,500 mg/day for 6 months (Parnetti et al., 1993)		
Uncontrolled studies		Choline alphoscerate dosed for the first 4 weeks as IM 1000 mg BID, then oral 1,200 mg BID for the next 20 weeks (Tomasina et al., 1991)	OL, choline alphoscerate 1,200 mg/day (Ban et al., 1991)
			Oral choline alphoscerate 1,200 mg/day for 6 months (Palleschi and Zuccaro, 1992)

^a^Including patients with Alzheimer’s disease or Parkinson’s disease. ^b^Each of the studies in this category included subjects with different types of cognitive impairment (of neurodegenerative and vascular origin). BID, twice a day; DB, double-blind; IM, intramuscular; IV, intravenous; OL, open-label; RCT, randomized controlled trial; SB, single-blind; vs, versus.

Compared with citicoline, choline alphoscerate had greater efficacy and more complete activity in an open-label study in patients with vascular dementia (Di Perri et al., 1991). Another study demonstrated improved efficacy with choline alphoscerate versus citicoline in patients with vascular dementia, as well as evaluating the effects of administering choline alphoscerate in 3-month cycles with a 3-month break between cycles (Muratorio et al., 1992). During off-treatment, the effectiveness on cognitive symptoms was maintained (Muratorio et al., 1992), suggesting that choline alphoscerate may also be administered in cycles, thus giving patients a break from treatment-associated burdens (e.g., cost, use of other drugs, excessive activation, psychomotor agitation, etc.).

Moreover, results of a recent study in Russia suggested that choline alphoscerate may help prevent development of dementia in patients with MCI at high risk of Alzheimer’s disease (Ponomareva et al., 2024). In this prospective, randomized study in 100 patients with amnesic type MCI, progression of cognitive deficits were reduced after 3 years of choline alphoscerate compared with no therapy (12.2% vs. 39.1%), and the conversion rate to Alzheimer’s disease was lower (8.2% vs. 26.1%) (Ponomareva et al., 2024). Another, multicenter, randomized, placebo-controlled study from South Korea assessed changes from baseline on the Alzheimer’s Disease Assessment Scale-cognitive subscale (ADAS-cog) to investigate the safety and effectiveness of choline alphoscerate for improving cognitive function in 100 overall healthy patients with MCI (Jeon et al., 2024). Treatment with choline alphoscerate significantly reduced the ADAS-cog score by 2.34 points after 12 weeks (*p* < 0.0001 vs. baseline and *p* < 0.05 vs. placebo).

Other researchers have shown that choline alphoscerate reduced conversion from MCI to Alzheimer’s disease dementia and vascular dementia, suggesting its value as an early intervention (Kim et al., 2025). Choline alphoscerate also lowered the risk of both ischemic and hemorrhagic stroke without increasing stroke risk, irrespective of dementia conversion.

### 5.2 Efficacy of choline alphoscerate on non-cognitive symptoms

Importantly, the most recent clinical studies demonstrate positive effects of choline alphoscerate on cognition and mood. A large, randomized study has shown stabilized or improved depression/apathy when choline alphoscerate is administered with donepezil compared with donepezil alone in patients with Alzheimer’s disease (Rea et al., 2015; Carotenuto et al., 2017; Carotenuto et al., 2022). In a different study, alphoscerate improved motivation compared with placebo in healthy volunteers (Tamura et al., 2021). Also, a systematic review and meta-analysis confirmed that addition of choline alphoscerate to donepezil significantly reduced behavioral symptoms and caregiver distress in patients with cognitive impairment (Sagaro et al., 2023).

In addition to the treatment of cognitive disorders, choline alphoscerate is indicated in the treatment of pseudo-, or subthreshold, depression in the elderly, as supported by recent guidelines issued by the Istituto Superiore di Sanità on the diagnosis and treatment of dementia and MCI (Istituto Superiore Di Sanità, 2024). These guidelines include choline alphoscerate to treat non-cognitive symptoms associated with dementia, and in particular apathy, in the light of the results obtained from the ASCOMALVA study (Traini et al., 2020; Istituto Superiore Di Sanità, 2024).

A recent comprehensive review of published preclinical and clinical literature confirmed the beneficial effects of choline alphoscerate in improving cognitive and behavioral conditions linked to cholinergic dysfunction and cognitive impairment in a range of mental conditions (Granata et al., 2025). The data suggest that choline alphoscerate may be an effective and safe therapeutic option to treat subthreshold depression in the elderly by improving mood regulation and motivation, reducing the risk of progression to major depressive disorders and enhancing quality of life (Granata et al., 2025).

### 5.3 Effects of choline alphoscerate on biomarkers of MCI and Alzheimer’s disease

Recent clinical studies have evaluated the effects of choline alphoscerate on various biomarkers in MCI and Alzheimer’s disease. Results of these studies have shown that addition of choline alphoscerate to donepezil reduces brain atrophy in patients with MCI or Alzheimer’s disease (Traini et al., 2020), and that electroencephalography changes may be a useful biomarker for therapeutic efficacy of choline alphoscerate in patients with MCI (Han and Chul Youn, 2022).

A randomized study evaluating the effects of choline alphoscerate on brain atrophy compared with placebo is ongoing (Carotenuto et al., 2024).

Recently published *in vitro* evidence suggests that cholinergic transmission is critical in suppressing glial proinflammatory cytokine production and enhancing intracellular Aβ_1–42_ clearance, synaptic plasticity and memory (Cantone et al., 2024; Munafo et al., 2024). Thus, using choline alphoscerate to modulate cholinergic transmission may be a useful therapeutic strategy for mitigating disease progression of inflammatory neurodegenerative disorders, such as MCI and Alzheimer’s disease.

## 6 Expert opinion on the use of choline alphoscerate

Based on our clinical experience, we advise choline alphoscerate be used in the following clinical scenarios:

- Primary or secondary cognitive disorders of the elderly, characterized by memory deficits, confusion and disorientation, decreased motivation and initiative, and reduced attention;
- Alterations of the affective sphere and senile behavior, including emotional lability, irritability and indifference to the surrounding environment; and
- Pseudodepression in the elderly.

The preferred schedule for choline alphoscerate administration is continuous, to ensure adequate concentrations of choline for enhancement of cholinergic tone. However, 3-monthly therapy cycles have demonstrated maintenance of drug effectiveness between the cycles.

Oral administration is the preferred option since it is less invasive; however, in patients where oral administration is not possible (e.g., in patients who are bedridden or care-dependent) choline alphoscerate can be administered intramuscularly. Also, in cases where initiation with a loading dose of choline alphoscerate is required, it is possible to start with intramuscular administration followed by transition to maintenance dosing with the oral formulation. Additionally, it is recommended that the dose be taken in the morning/early afternoon, in order to not interfere with night-time rest. The total daily dose of 1,200 mg of choline alphoscerate can be administered as 2 doses of 600 mg or 3 doses of 400 mg. This dosage is necessary for the patient to ensure adequate drug levels throughout the 12 h of wakefulness, while administration of the last dose by early afternoon avoids excessive cholinergic stimulation, and therefore activation/agitation, which could interfere with sleep.

## 7 Conclusion

The efficacy of choline alphoscerate on cognitive function is well established in patients with MCI, Alzheimer’s disease or cognitive impairment of vascular origin. However, emerging evidence suggests that the administration of this cholinergic precursor may also be useful as a preventive therapy against development of Alzheimer’s disease in patients with MCI and for the treatment of non-cognitive symptoms in patients with MCI. Further research is warranted.
