# Correction: Choline alphoscerate: insights between acquired certainties and future perspectives

**DOI:** 10.3389/fnagi.2026.1744201

**Published:** 2026-01-29

**Authors:** Giovanni Biggio, Claudio Mencacci

**Affiliations:** 1 Department of Life and Environmental Sciences, University of Cagliari, Cittadella Universitaria di Monserrato, Cagliari, Italy; 2 Institute of Neuroscience, CNR, Cittadella Universitaria di Monserrato, Cagliari, Italy; 3 Department of Neuroscience and Mental Health, ASST Fatebenefratelli Sacco, Milan, Italy

**Keywords:** aging, choline alphoscerate, cognitive dysfunction, mild cognitive impairment, sleep disorders

## Abstract

**Figure:**
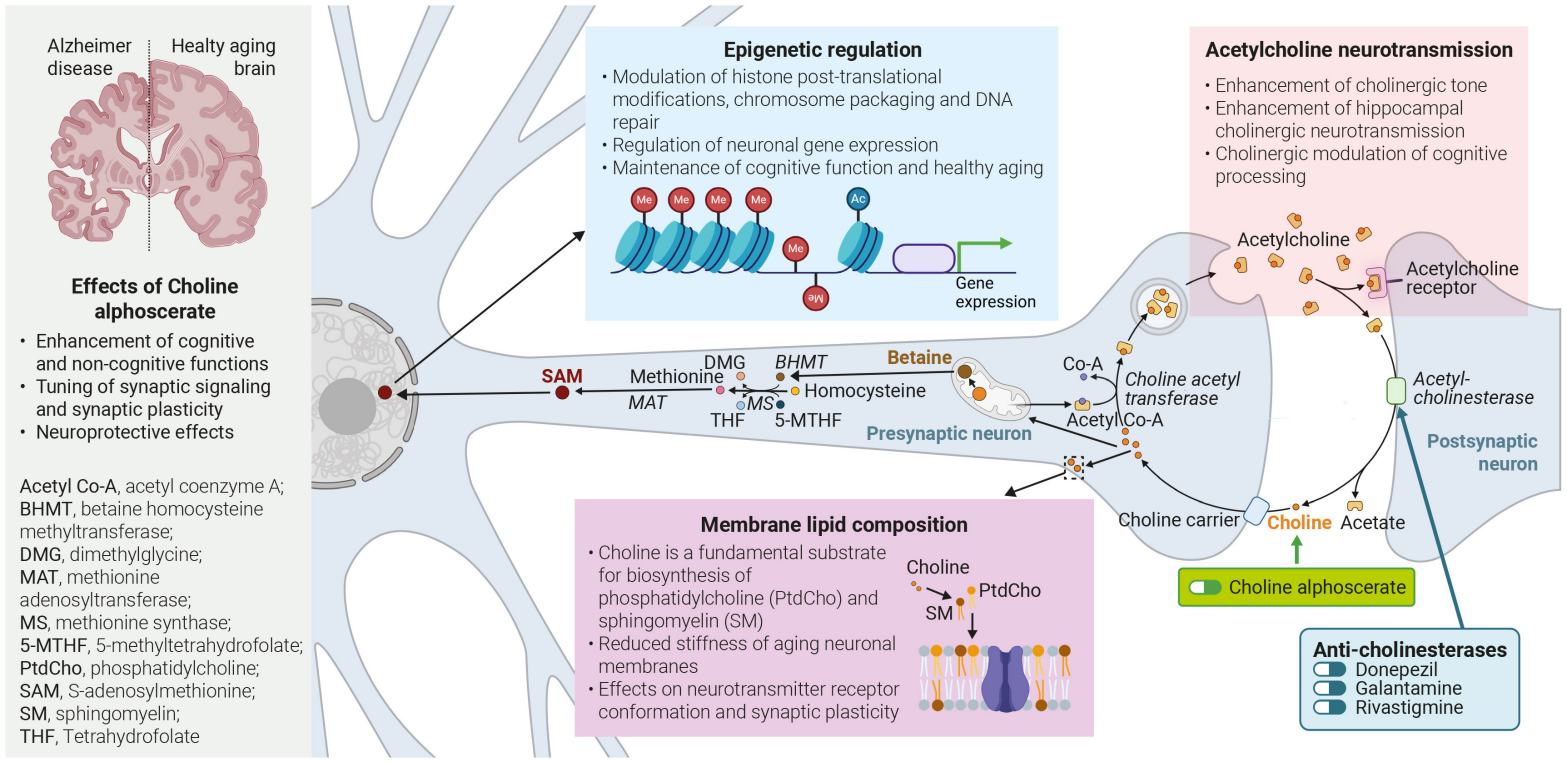

The graphical abstract of the published article was erroneously omitted. The graphical abstract appears below.

The original version of this article has been updated.

